# Discovery of Anesthesia

**Published:** 1900-12-15

**Authors:** 


### DISCOVERY OF ANESTHESIA.

BY DR. HORACE WELLS.

At the February meeting (1894) of the Odontological Society of Pennsylvania, the statement was made, that in the coming December would occur the fiftieth anniversary of the discovery of anesthesia, by Dr. Horace Wells. A committee was then and there appointed to take action for the celebration of that great event.

This committee proceeded with its work and at the August meeting of the American Dental Association, 1894, the subject of such a commemoration was presented.

A committee of nine was appointed by that body to take preliminary steps for the carrying out of the proposition. This committee at a later session presented a report giving in detail the program of procedure, for the purpose indicated.

In this report the committee recommended that an organization committee of nine, an executive committee of eleven and a general committee of fifty be appointed for carrying out the work.

These committees did their work well and the fiftieth anniversary of the discovery of the anesthetic properties of nitrous oxid, by Dr. Horace Wells, of Hartford, Conn., was observed in Philadelphia, December 11, 1894. These proceedings have been printed in a separate volume for preservation, and as a memorial of the part dentistry has taken in the discovery of anesthesia.

On this occasion a public meeting was held in the afternoon in Association Hall, and a dinner at the Union League in the evening.

Immediately after the celebration exercises the question of securing a bronze bust of Dr. Wells was suggested. Action was taken upon the suggestion, and eleven hundred and five dollars were contributed, enabling the committee to place in the library of the Army and Navy Medical Museum, in Washington, a beautiful bronze bust. In opening the exercises, remarks were made by Profs. Fillibrown, of Boston, and J. Y. Crawford, of Nashville, which was followed by the reading of a paper by Dr. Fillibrown on the "History of Anesthesia."

This address is a masterly one, presenting the salient points of this subject in clear and concise terms.

At the close of Dr. Fillibrown's address, Prof. J. E. Garretson, of Philadelphia, read a paper giving a glowing account of the benefits of anesthesia to mankind. This paper, though not read in full by Dr. Garretson, is presented in this volume as a whole. It is one of exceeding interest, and in a striking manner emphasizes the value of nitrous oxid anesthesia. Following Dr. Garretson's address, Dr. G. Q. Coleton, of New York, read a paper giving his experience in connection with the extraction of teeth. Certainly no one in this country has had more experience in the use of nitrous oxid, than Dr. Coleton. He was present at the first experiment, and had continued its use for the extraction of teeth, up to the time of this event. His paper threw much light upon the incidents connected with its early use.

After the reading of this paper a discussion of the whole subject was had by those present.

The persons taking part in this discussion were, Doctors Darby and Truman, of Philadelphia; L. D. Shepherd, of Boston; Jas. Wm. White, of the University of Pennsylvania; Senator Hawley, of Hartford; Col. McClure, of Philadelphia, and quite a number of others.

This volume contains 124 pages and gives a presentation of this subject nowhere else to be found in our literature, at least in so attractive a form. It should be in the library of every dentist who desires to keep abreast with his chosen profession. The book is beautifully prepared by the Patterson & White Co., Philadelphia. TAFT.

